# Bilingual Language Control and General Purpose Cognitive Control among Individuals with Bilingual Aphasia: Evidence Based on Negative Priming and Flanker Tasks

**DOI:** 10.1155/2014/679706

**Published:** 2014-05-07

**Authors:** Tanya Dash, Bhoomika R. Kar

**Affiliations:** Centre of Behavioural and Cognitive Sciences, Senate Hall Campus, University of Allahabad, Allahabad-211002, India

## Abstract

*Background*. Bilingualism results in an added advantage with respect to cognitive control. The interaction between bilingual language control and general purpose cognitive control systems can also be understood by studying executive control among individuals with bilingual aphasia.* Objectives*. The current study examined the subcomponents of cognitive control in bilingual aphasia. A case study approach was used to investigate whether cognitive control and language control are two separate systems and how factors related to bilingualism interact with control processes.* Methods*. Four individuals with bilingual aphasia performed a language background questionnaire, picture description task, and two experimental tasks (nonlinguistic negative priming task and linguistic and nonlinguistic versions of flanker task).* Results*. A descriptive approach was used to analyse the data using reaction time and accuracy measures. The cumulative distribution function plots were used to visualize the variations in performance across conditions. The results highlight the distinction between general purpose cognitive control and bilingual language control mechanisms.* Conclusion*. All participants showed predominant use of the reactive control mechanism to compensate for the limited resources system. Independent yet interactive systems for bilingual language control and general purpose cognitive control were postulated based on the experimental data derived from individuals with bilingual aphasia.

## 1. Introduction 

Bilingualism and cognitive control are two widely studied phenomena. Numerous studies have examined the interaction between bilingualism and cognitive control using different methodologies and paradigms [1–11]. Juggling two or more languages makes our brain more flexible [10]. The bilingual advantage has been well established not only with respect to studies comparing monolingual and bilingual individuals [6, 12–14] but also among different bilingual groups [6–9]. Interestingly, a review by Adesope et al. [15] suggested that different aspects of bilingualism influence distinct levels of cognitive control mechanisms. Moreover, several cognitive outcomes may be attributed to bilingualism, including increased attentional control, working memory, metalinguistic awareness, and abstract and symbolic representational skills. Researchers have differentiated between bilingual language control and domain general cognitive control [2, 16]. Miller and Cohen [17] proposed that to provide top-down support to language control, processes such as attention, working memory, response selection, and inhibition function as different manifestations of domain general cognitive control. Moreover, bilingual language control (bLC) may not be a subsidiary to domain general cognitive control [16]; however, bLC may still show some overlap with domain general control mechanisms [2, 18].

Different frameworks have been suggested to study the interaction between bilingualism and cognitive control. A few studies have examined the interaction between bilingualism and cognitive control in the context of bilingual aphasia [18–20]. Aphasia is a language impairment caused by brain damage. Language-related deficits are well explained in the literature targeting auditory comprehension, naming skills, spontaneous speech, and repetition as well as reading and writing skills. There are lines of evidence supporting the presence of cognitive impairment in individuals with aphasia [19, 21]; however, most of these studies refer to the independence of deficits in language skills and other cognitive processes. In one such study, Helm-Estrabrooks [22] argued that clinicians cannot predict the relative integrity of other domains of cognition on the basis of language deficits observed in aphasic patients. Another group of researchers [23] discussed the implications of different aspects of cognition in language-related treatment approaches. They employed a global aphasic neuropsychological battery (nonlinguistic tests) and a test of auditory comprehension. The battery of tests assessed attention, memory, intelligence, visual recognition, and nonverbal auditory recognition. The authors concluded that the score on this battery was independent of spoken language comprehension.

Communicative success among individuals with aphasia may be dependent on the integrity of the executive functions that allow us to plan, sequence, organise, and monitor goal-directed activities in a flexible manner. While emphasising the role of executive functions in communicative processes, Helm-Estrabrooks and Ratner [24] suggested that deficits in executive functions may result in a failure in the generalisation of skills, which are similar to those learned in therapy sessions to those learned in everyday life situations. Similarly, Purdy [19] conducted a study investigating the efficiency, speed, and accuracy of individuals with aphasia while performing executive function tasks (i.e., Porteus Maze Test, Wisconsin Card Sorting Test, and Tower of London). Their deficits were predominantly related to cognitive flexibility and, to a lesser extent, planning.

Until recently, cognitive impairments in aphasia were studied in isolation; however, this can be well explored through empirical research by examining the underlying mechanisms that manifest as cognitive impairments. A study by Penn et al. [25] supports this notion of bilingual advantage, in which they compared monolingual and bilingual individuals with aphasia. If bilingualism is a cognitive advantage, then bilingual aphasics may demonstrate a faster rate of language recovery compared to monolingual aphasics. However, bilingual aphasics exhibited pathological code switching and code mixing behaviours. In one of their studies, Abutalebi and Green [2] highlighted the need to investigate the performance of bilingual aphasics on a range of control tasks. They suggested that individuals with parallel recovery may demonstrate problems with control without having problems related to language interference.

In addition to the language processing deficits evident among individuals with aphasia, there are subtle cognitive-communicative deficits, which are not due to the faulty language processing system but may be due to general problems in resolving conflict. Green et al. [26] reported that there are two distinct control-related impairments, one for naming and another for control. Green and colleagues compared two bilingual aphasics who demonstrated a parallel form of recovery to a similar extent. However, their performance on three explicit control tasks indicated that different control mechanisms were involved in recovery. One of the patients showed an impaired verbal, but spared nonverbal control, whereas the other patient demonstrated deficits in the selection of the manual response. Thus, two separate circuits may exist for naming and control, and the recovery patterns may be dependent on damage to these control circuits [2].

According to Abutalebi et al. [18], language control and cognitive control mechanisms may act as the primary determinants of cognitive-linguistic recovery in aphasia. This is because the effect of treatment is dependent on the integrity of the naming and control pathways as previously described by Abutalebi and Green [2]. To understand this distinction between naming and control networks, Abutalebi et al. [18] studied the neural correlates of selective language therapy in a Spanish-Italian bilingual aphasic in a longitudinal study consisting of three time points. An improvement in naming performance was evident in the naming network only. Another study [20] emphasised the role of the dorsal anterior cingulate in both language control and while resolving nonverbal conflict. Using a combined functional and structural neuroimaging method, a structural overlap between the two networks (i.e., naming and control) was demonstrated. These studies demonstrated that there was a dissociation as well as an overlap between the mechanisms that were involved while resolving verbal and nonverbal conflict.

Conflict resolution tasks involve two modes of control mechanisms, namely, proactive and reactive controls. The proactive mode of control is prospective or future-oriented, helping to prepare the cognitive system for upcoming events via the predictive use of context. Reactive control is retrospective, responding to the presence of salient events by engaging control only when it is needed, via the reactivation of previously stored information [27]. In the context of bilingualism, these two modes of control might be operating in cases of conflict and during increasing demand on the inhibitory control system while using activation-suppression mechanisms. Thus, this study aimed to address the relationship between language control and general purpose cognitive control with respect to the recruitment of proactive and reactive control mechanisms among bilingual aphasics.

The current study was designed to test patients on a range of executive function tasks that bear on the circuits implicated in language control and general purpose cognitive control. The specific objective was to examine differences in performance across executive control tasks with linguistic and nonlinguistic stimuli. Flanker and negative priming paradigms were employed to show the distinction in performances with different cognitive outcomes between the two paradigms. One way to understand how control mechanisms are recruited by bilingual aphasics is to examine the slow and fast trials, which indicate the use of reactive and proactive control mechanisms, respectively. Special emphasis was placed on accuracy, efficiency, and speed-related measures, unlike previous studies, which focused on one of the three aspects of performance. Predominant involvement of reactive control compared to proactive control mechanisms in the context of both linguistic and nonlinguistic stimuli was expected. Differences in performance were expected with respect to negative priming and flanker effects in the two paradigms, indicating variability in different control processes. Thus, the present study helps to understand the interactive yet independent control mechanisms in the clinical population, particularly in language disorders, such as aphasia, which provide the appropriate context to examine the relationship between bilingualism and cognitive control. In addition, such an investigation also helps to understand the broad cognitive-linguistic mechanisms that underlie a disease process and its recovery patterns.

## 2. Method

### 2.1. Screening Measures

#### 2.1.1. Language Background Questionnaire [28]

This questionnaire was employed to collect information on the languages in use, frequency of use, self-reported proficiency, and the linguistic environment at home, work, and so forth. Domains assessed in the questionnaire included acquisition history (age of acquisition and at what age the subject became fluent), contexts of acquisition (modality: oral/written/both; environment of acquisition: informal/formal/both), language use (%), language preference (1–3 rating scale; where 1 = never, 2 = sometimes, 3 = most of the time), and the proficiency rating on different tasks (a 0–10 rating scale was provided for each descriptive task, for example, asking for directions, counting up to 100 in both languages, and so forth, which resulted in a composite score for proficiency). Apart from these questions, a contribution of various other factors, such as the use of language with family, friends, extended family, and neighbours was assessed by asking the participants to name the language predominantly used in different contexts. The participants also indicated the medium of instruction and self-reported proficiency level in the domains of speaking, understanding, reading, and writing (1–5 point rating) (see Table 1 for language background information for all the participants).

#### 2.1.2. Picture Description Task

This test is a subtest of the language proficiency test [29]. In this task, the participants were instructed to carefully describe a picture by focusing on the overall theme of the picture along with the individual items in that particular picture. A grand rubric score was calculated by summing the scores on the following aspects: overall impact and achievement of purpose (whether the participant establishes the main idea), organisation and techniques (coherence and cohesion, method of organisation), and mechanics (focusing on grammar, pronunciation, presence of pause). Pictures were selected from the Western Aphasia Battery [30] and Boston Diagnostic Aphasia Examination [31] for L1 and L2, respectively. A total score of 18 could be achieved by each participant (Table 5 presents the scoring method).

#### 2.1.3. Western Aphasia Battery [30]

WAB is a tool used to assess language functions in adults and discerns the presence and type of aphasia. Four major components of the aphasia quotient are spontaneous speech, auditory verbal comprehension, repetition, and naming.  Table 3 presents the scores obtained on each of the subtasks in WAB for the four participants.

#### 2.1.4. Aphasia Severity Rating Scale [31]

This is a severity rating scale that is often used in clinical routine as well as in scientific studies. Administration of this scale takes 5–15 minutes and is very simple to perform. It is a 5-point rating scale where the communication profile is described and based on the clinical observation that one can make a judgment about severity.  Table 2 presents the ratings indicating the severity of aphasia for each of the four participants.

### 2.2. Participants

We report data from four male bilingual right-handed individuals with aphasia. English was the second language for all the participants. L1 was Telugu for two participants and Hindi for the other two participants. All four participants were considered for the current study based on the following inclusion criteria: (a) diagnosis of aphasia based on the Western Aphasia Battery [30], (b) above chance level performance on the experimental tasks, (c) average level of intellectual functions on Raven's Coloured Progressive Matrices test (as a subtask in WAB), (d) being able to perform the picture description task from the test of language proficiency, which provides a composite rubric score [29], (e) similar degree of impairment in L1 and L2, and (f) postmorbid daily usage of both languages in the speaking/understanding domain as well as in the reading/writing domain. These criteria were met using the subjective and objective measures mentioned above as well as the clinician's report.

All participants showed parallel recovery based on their performance on the language skills tasks as well as the self-reported information on the language background questionnaire [28]. All four participants were highly educated and were able to perform the activities of daily living. The experimental and language proficiency tasks were performed on the same day with many rest pauses (see Table 2 for a summary of the demographic information of all the participants).

#### 2.2.1. Participant 1

CR was a 33-year-old, right-handed Telugu-English bilingual male, who was a banker prior to his illness. He had resumed his work on a part-time basis a few days from the time of his current evaluation. CR reported a complaint of a loss of speech due to a postmeningitis squeal. It had resulted in diffuse damage to the left frontal and parietal regions as per the clinician's report. CR was initially diagnosed with Broca's aphasia and is currently diagnosed with anomic aphasia. CR had been undergoing speech and language therapy for 15 months prior to the time of his current evaluation on a regular basis. On the WAB subtests, his language skills were affected in all four WAB subtasks; namely, spontaneous speech, auditory verbal comprehension, repetition, and naming. His repetition subtask score was below the 50th percentile, and his naming subtask score was at the 50th percentile level. His auditory verbal comprehension skills were better than the rest of his skills. Performance on the picture description task in both languages showed an impairment at the discourse level with rubric scores of 11 and 9 for L1 and L2, respectively (maximum score of 18). His spontaneous speech showed the presence of both circumlocutions and paraphasic errors (semantic). Language switching or mixing was neither observed nor reported.

#### 2.2.2. Participant 2

MMH was a 34-year-old right-handed male and presented with a history of cerebrovascular disease. He was diagnosed with aphasia and was undergoing therapy. He was initially diagnosed with global aphasia and is currently diagnosed with anomic aphasia. He had a lesion in the left cerebral hemisphere involving the insular cortex, frontal, and frontoparietal region, which was suggestive of an infarct in the left middle cerebral artery territory. MMH had been undergoing speech and language therapy for 17 months prior to the time of his current evaluation. He was unemployed at the time of his current evaluation and had been undergoing speech and language therapy as well as physiotherapy and occupational therapy due to right hemiparesis. On the WAB, his spontaneous speech was greatly affected, with a score in the 70th percentile, whereas his scores were greater than the 80th percentile on rest of the tasks, namely, the auditory verbal comprehension, repetition, and naming subtasks. His spontaneous speech showed problems in fluency as well as in speech initiation. Performance on the picture description task in both languages resulted in a composite rubric score of 14 and 12 in Hindi and English, respectively. No significant problems were observed in language selection.

#### 2.2.3. Participant 3

SC was a 35-year-old right-handed male who presented with a history of head trauma, which resulted in a subdural hematoma in the left hemisphere involving the frontal regions. At the time of the current evaluation, SC was actively participating in the family business. His initial diagnosis was global aphasia, and his current diagnosis was Broca's aphasia. He had been regularly attending speech and language therapy sessions for three months since the injury. He demonstrated difficulties in the naming (26%) and repetition (13%) subtasks on the WAB, similar to Participant 1. Circumlocution and paraphasia were also observed more in English than Telugu. His auditory comprehension skills were better than the rest of the subtasks on the WAB with 94% accuracy. There was a difference in his performance between L1 and L2 on the picture description task. He showed a greater impairment in L2 compared to L1 with composite rubric scores of 11 and 6 for L1 and L2, respectively.

#### 2.2.4. Participant 4

MU, a 59-year-old right-handed male was working at a higher administrative position at an academic institution prior to his stroke. He had experienced an ischemic stroke involving the MCA territory, which caused a typical lesion in the left frontal areas as well as white matter lesions. He presented with hemiparesis and an inability to speak. His initial diagnosis was global aphasia, and his current diagnosis was anomic aphasia. He had undergone therapy for a period of 20 months. On the auditory verbal comprehension subtask, his score was more than 90% similar to the scores of Participants 2 and 3. However, his WAB profile matched more with Participant 2. He showed a similar performance in L1 and L2 on the picture description task with scores of 14 and 12, respectively (see Table 3 for scores on the WAB for all participants).

### 2.3. Control Tasks

#### 2.3.1. Nonlinguistic Negative Priming Task

Negative priming describes the phenomenon of a prolonged reaction time (RT) and/or a greater number of errors when the participants have to respond to a target that was ignored in the preceding trial [32, 33]. In this task, the participants were required to process pairs of trials that were structured according to a prime-probe schema. Two picture stimuli (line drawings of animate or inanimate objects) were displayed in the form of overlapping pictures in shades of grey on both trials: one picture was the target in which the participants must respond and the other was the distracter, which must be ignored. In the present experiment, the participants were required to respond to one of the shades of grey (dark grey with the RGB coordinates 60, 60, 60, or light grey with the RGB coordinates 157, 157, 157) by suggesting the identity of the picture as being animate or inanimate.

The stimuli were presented on a 17′′ monitor in a quiet dimly lit room. The stimuli appeared at the centre of the screen, which measured within the frame of 106 pixels ∗ 52 pixels. The horizontal and vertical resolutions were fixed at 71 dpi. The participants were seated comfortably at a distance of 60 cm from the computer monitor. The experiment was programmed using E prime version 2.0 to record the reaction time and accuracy of each trial. Each trial began with a fixation point for a duration of 400 milliseconds (ms) followed by a prime-probe stimuli, which was presented on a white background for a duration of 500 ms. These stimuli were each separated by a 300 ms blank screen. During the probe trial, the blank screen remained until the response or 3000 ms, whichever came first, and then the next trial began with a fixation point. The participants were required to press the right arrow key for animate and left arrow key for inanimate targets using the first and second fingers of their dominant hand. They were instructed to respond as quickly and as accurately as possible.

The experiment consisted of a total of 180 trials with 60 trials for the attended repetition condition, 60 trials for the ignored repetition condition, and 60 trials for the control condition. The attended repetition measured the facilitation effect in performance. In such a condition, the picture being attended in the prime trial was attended again on the probe trial, resulting in faster reaction times compared to the control and ignored repetition conditions. The ignored repetition measured the inhibitory effect on performance. In such a condition, the picture being ignored in the previous trial was attended in the probe/current trial, resulting in an increase in reaction time (slowing of the response) compared to the other two conditions.Thecontrol condition acts as a baseline measure for the experiment in which the pictures (two overlapping pictures) in the prime trial were different from the probe trial. Thus, there was no effect of priming, whether positive or negative priming. The accuracy and reaction times were recorded for each condition for all four participants. The analysis was performed based on these three conditions. A linguistic counterpart of the negative priming task with a similar design could not be performed because it involved perceptually complex stimuli with overlapping words, and these stimuli appeared to be difficult for individuals with aphasia during the pilot phase of the study.

#### 2.3.2. Flanker Task with Linguistic and Nonlinguistic Stimuli

The flanker task is a response inhibition task that is used to assess the ability to suppress responses that are inappropriate in a particular context. The flanker paradigm was originally introduced as a way to study the cognitive processes involved in the detection and recognition of targets in the presence of distracting information or noise [34]. In the present study, Eriksen's Flanker task [34] was employed to measure executive control to examine conflict resolution with two comparable tasks using linguistic and nonlinguistic stimuli. To introduce a conflict resolution component, the central arrow is “flanked” by congruent or incongruent stimuli. The target is flanked on either side by two arrows in the same direction (congruent condition) or in the opposite direction (incongruent condition). On some trials, the target is flanked by neutral flankers (neutral condition), which were neither similar to the target nor to the flankers in the incongruent condition. The same conditions were used in the current study. Both the target and flankers appeared simultaneously. The participants were required to respond to the direction of the central target arrow, which could be facing in the same direction as the flankers (congruent condition) or in the opposite direction compared to the flankers (incongruent condition). There was also a neutral condition, which consisted of a central target arrow that faced either left or right with dashes as the flankers on either side of the target, thus resulting in a no conflict condition. Each trial began with a fixation cross for 400 ms followed by the stimuli (target and flankers), which were presented for 500 ms followed by a blank screen that stayed until the response or 3000 ms, whichever came first. The participants were required to respond by pressing the right arrow key on the keyboard if the target was facing towards the right, and the left arrowkeyif the target was facing towards the left. There were 180 trials in total, with 60 trials in each condition. There were approximately 30 practice trials in the beginning of the session prior to starting with the main experimental trials.

The linguistic version of the flanker task in different language pairs was designed with letters from the two languages (L1 and L2) known to each participant (Hindi-English and Telugu-English). This task was based on the standard flanker task, but with two flanker compatibility conditions (congruent and incongruent). It did not include the neutral condition because it would have resulted in an unequal number of incongruent trials for both the languages (because the incongruent condition also had two levels). The number of flankers was the same as the nonlinguistic version. The only addition was the presence of two types of incongruent trials: those with within-language incongruence (HHSHH) and those with across language incongruence (HH
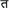
HH).

We determined the appropriateness of the stimuli (letters) in the pilot study using normal healthy participants. Each trial began with a fixation cross for 400 ms followed by the target letter flanked by congruent (flanking letters were the same as the target letter) or incongruent flankers (flanking letters were different from the target letter). The stimuli were presented against a white background for 500 ms followed by a blank screen. The blank screen remained until the response or 3000 ms, whichever came first, and then the next trial began with a fixation cross. The participants were required to press the right arrow key for “H,” left arrow key for “S,” up arrow key for 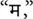 and down arrow key 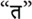 for the flanker task with stimuli in Hindi and English languages. A similar design was used for the Telugu-English version of this task. A total of 360 trials were presented, with 120 trials in each condition, which were congruent, incongruent within a language, and incongruent across language. These conditions were equally divided for both the languages. The response level inhibition resulted in slowing of the responses on the incongruent trials and varied as a function of language. Eriksen's flanker task has also been reported for linguistic stimuli, but only with one language [34].

In both versions of the flanker task (linguistic and nonlinguistic), the stimuli were presented on a 17′′ monitor with a refresh rate of 85 Hz in a quiet and dimly lit room. The participants were comfortably seated at a distance of 60 cm from the computer monitor. In the linguistic version of the task, the array of letters appeared on the centre of the screen within the frame of 140 pixels ∗ 45 pixels, whereas in the nonlinguistic version arrows appeared within the frame of 135 pixels ∗ 25 pixels. The experiment was programmed using E prime version 2.0 to record the reaction time and accuracy for each trial.

## 3. Results

The current study focused on the performance patterns of each participant on the cognitive control tasks, and the subjective and objective measures of language proficiency. The data obtained with the language background questionnaire and the composite rubric scores on the picture description task are provided in Table 1. Data based on the performance of each participant on the respective cognitive control tasks are shown in Table 4. We discussed the results based on the variations in the performance of each participant on the cognitive control tasks as well as their language background information. Statistical inference was generated via visual analysis of the data (mean RT scores as well as CDF plots of different conditions) for each participant for each specific experiment. Correlation analysis was performed to test the relationship between objective and subjective task performance. The variability in a single case study method was controlled using experimental tasks and tools for language proficiency, which have been well adapted for Indian conditions. Negative priming and the flanker paradigm are well established paradigms employed across populations; thus there is a limited chance of variability because of the measurement instrument. This language history questionnaire has been employed in an Indian population [29, 35] in both qualitative and quantitative bilingual studies.

The cumulative frequency distribution was employed as an important tool for the interpretation of individual data to examine the performance patterns of each task across the four participants. To analyse the reaction time data, the cumulative frequency data were used to gain insight into how often a specific phenomenon was either below or above a specific value. The RT distributions were computed using the cumulative distribution function (CDF) in MATLAB. We examined the RT-based differences at the 5th percentile (fast trials) and 95th percentile (slow trials) across conditions for each participant. In a few instances, a different range of percentiles was used to indicate patterns in the performance of specific experimental conditions. Slow and fast reaction times were used as measures of the proactive and reactive modes of control. Slow trials are known to reflect the involvement of the reactive control mechanisms and fast trials are known to reflect the involvement of proactive control mechanisms [27].

The results are discussed with respect to the patterns in the performance on each experimental task across the four participants. In this study, the primary objective was not to compare the performance of the four participants but to illustrate the variations in each participant's performance across tasks and across conditions (experimental manipulations) within a task.

### 3.1. Negative Priming Task with Nonlinguistic Stimuli

All four participants performed the negative priming task with a good overall accuracy except for SC who showed a below chance level performance on one of the tasks. However, variations in performance across participants were observed with respect to the engagement of proactive and reactive control mechanisms as revealed by the RT distributions on the 5th and 95th percentiles.

**Figure 1:**
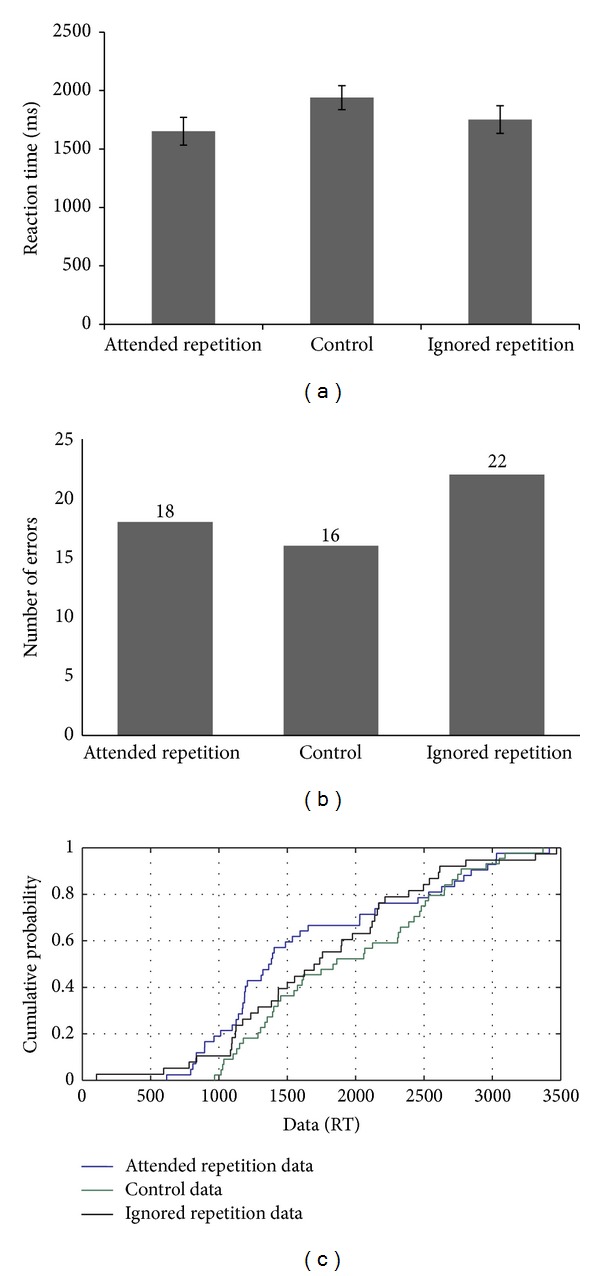
(a–c): Reaction time data, error analysis, and CDF plot based on the performance of CR on the negative priming task.

CR's performance on the negative priming task with superimposed line drawings of objects (with reaction times as a measure of performance) suggests the presence of a facilitation effect in the absence of a negative priming effect (see Figure 1(a)). However, error analysis showed a greater number of errors for the ignored repetition trials compared to the control condition, suggesting the presence of a negative priming effect (see Figure 1(b)). In addition, CDF plots further supported these results. CDF curves showed facilitation or a positive priming effect more prominently in the fast trials (i.e., 5th percentile) (see Figure 1(c)). This effect was persistent throughout the distribution except at the 95th percentile level (i.e., slow trials) where the distribution appeared to be very similar across conditions. These results indicated that when the time to respond to a target is less, then the facilitation effect is greater compared to when the time available is more. These results indicated the presence of proactive control in a minimal conflict condition (i.e., attended repetition condition). Similarly, ignored repetition reaction times were faster compared to the control condition throughout the distribution except for the 95th percentile level.

Taken together, these results indicated that although the overall mean RTs showed an absence of a negative priming effect, the negative priming or persistent inhibitory effect surfaced only on the slow trials, when the time available was more, indicating a dependence on the reactive control mechanism. The proactive control mechanisms appeared to be compromised. However, because the error analysis showed a negative priming effect with a greater number of errors on the ignored repetition trials compared to the control trials, this in itself may be the reason why fast trials did not show a negative priming effect. Thus, when the participant takes less time during a more demanding condition (i.e., ignored repetition), it may result in a greater number of errors.

**Figure 2:**
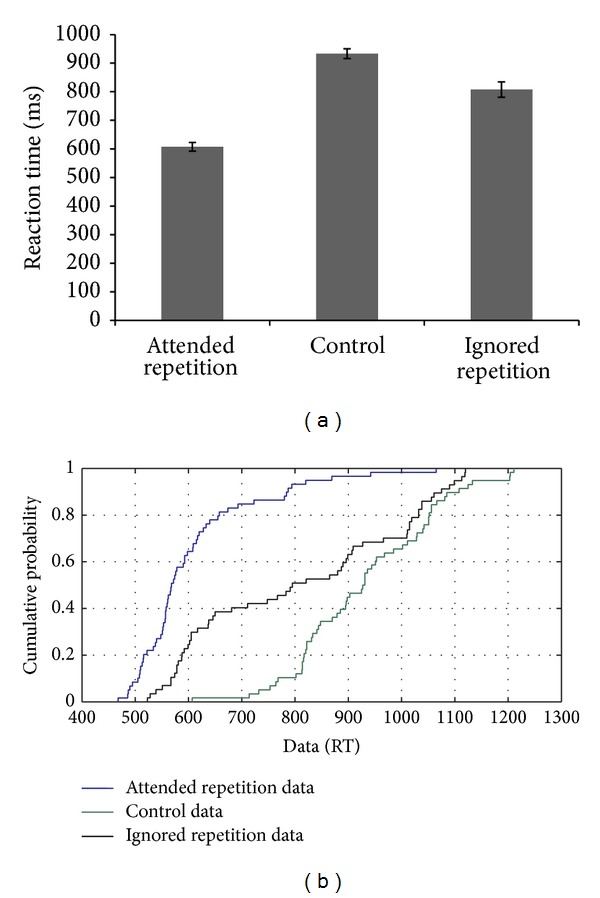
(a-b): Reaction time data and CDF plot based on the performance of MMH on the negative priming task.

MMH's performance on the negative priming task showed facilitation or a positive priming effect, and a negative priming effect was not observed (see Figure 2(a)). CDF analysis only showed the presence of a facilitation effect and the absence of a negative priming effect based on the observation that there was no difference between the RT distributions for the ignored repetition condition and control condition (see Figure 2(b)). Furthermore, the facilitation effect was greater on the slow trials compared to the fast trials. An absence of an inhibitory effect was observed in both the fast and slow trials. The results based on MMH's performance indicated a potential dependence on reactive control mechanisms and showed a partial correspondence with the RT distributions observed on the nonlinguistic flanker task, as discussed later in this section.

**Figure 3:**
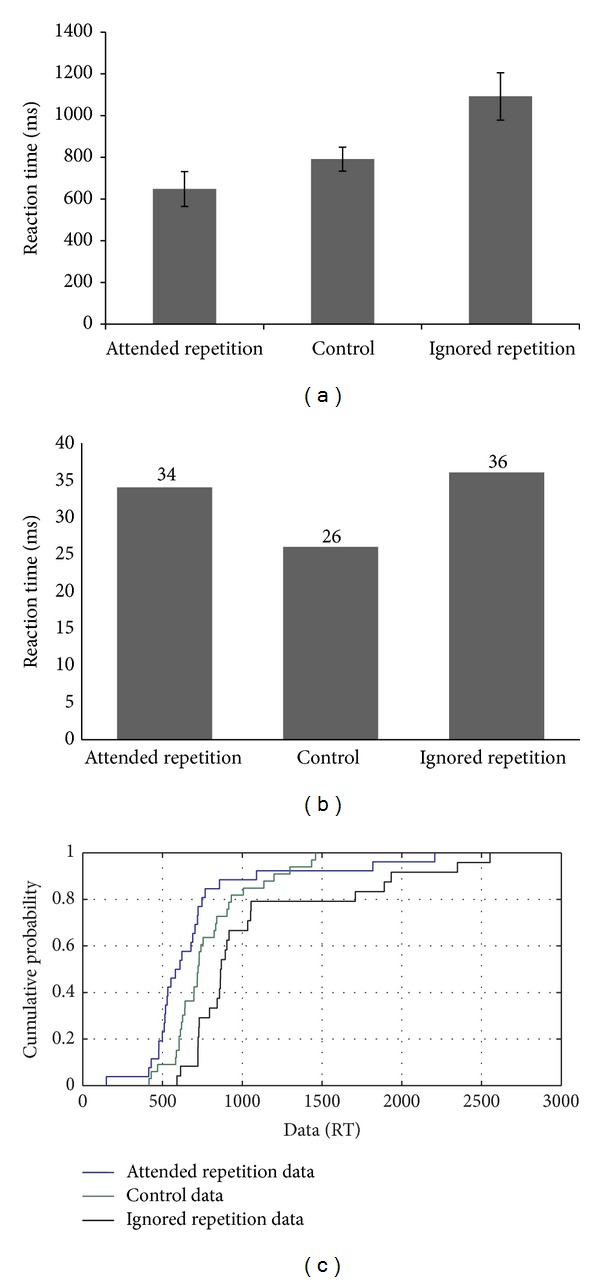
(a–c): Reaction time data, error analysis, and CDF plot based on the performance of SC on the negative priming task.

The third participant, SC, performed at a below chance level on the negative priming task. However, interestingly, his performance (RTs) indicated both facilitation and inhibitory effects (see Figure 3(a)). Error analysis suggested the presence of an inhibitory effect with a greater number of errors on the ignored repetition condition compared to the control and attended repetition conditions, and an absence of the facilitation effect (no difference in errors between the attended repetition condition and control condition) (see Figure 3(b)). CDF plots also showed a uniform distribution for all three conditions (attended repetition < control < ignored repetition) showing no variations in performance with respect to the fast and slow trials across conditions (see Figure 3(c)). However, visual inspection of the CDF plots suggested a greater inhibition on the slow trials compared to the fast trials.

**Figure 4:**
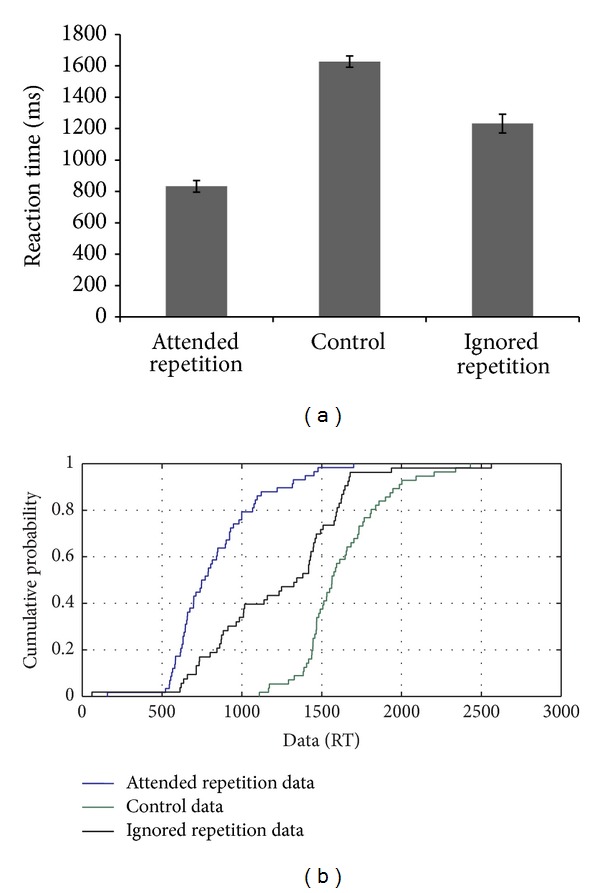
(a-b): Reaction time data and CDF plot based on the performance of MU on the negative priming task.

MU's performance on the negative priming task with nonlinguistic stimuli, similar to CR and MMH, showed the presence of a facilitation effect and the absence of a persisting inhibitory effect (see Figure 4(a)), which was translated in the same manner in the CDF analysis. However, for the ignored repetition condition, there were variations in performance throughout the distribution compared to the control condition. CDF plots indicated that the inhibitory or negative priming effect only appeared on the slow trials, indicating the involvement of reactive control mechanisms (see Figure 4(b)).

Thus, the performance of all the participants on the negative priming task primarily reflected the involvement of reactive control mechanisms. Proactive control mechanisms appear to be affected with respect to the persistent inhibitory effects as indicated by the subjects' performance on the negative priming task.

### 3.2. Flanker Task with Nonlinguistic Stimuli

All of the participants performed the flanker task with nonlinguistic stimuli with good accuracy except for SC, who showed less accuracy but at an above chance level.

**Figure 5:**
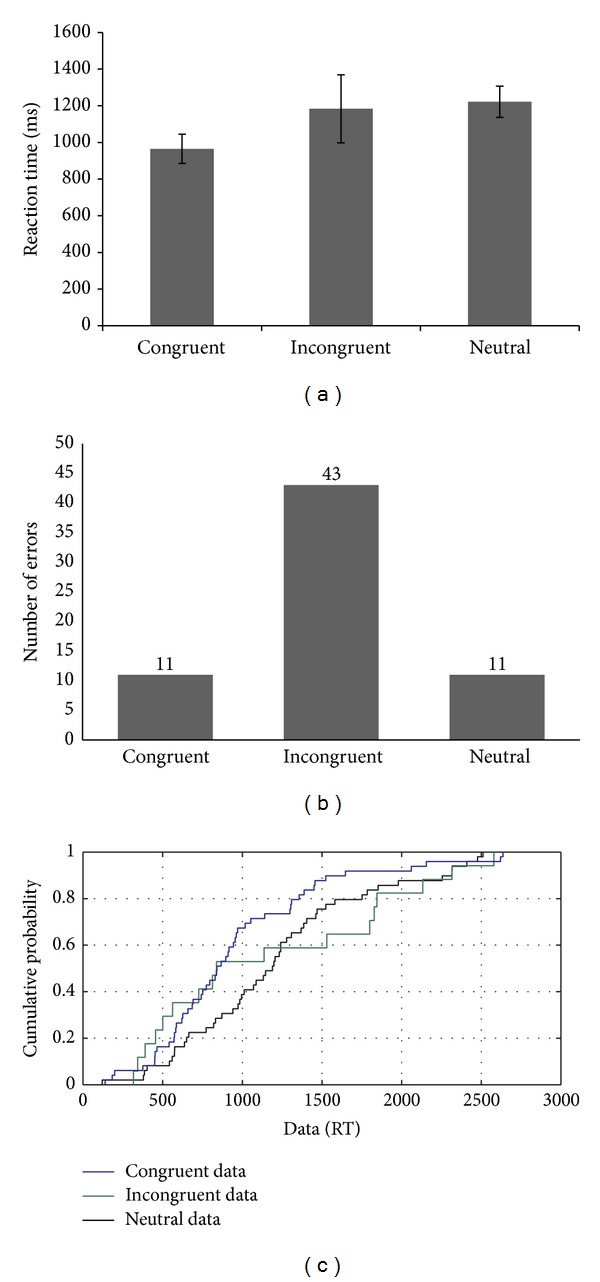
(a–c): Reaction time data, error analysis, and CDF plot based on the performance of CR on the flanker task with nonlinguistic stimuli.

CR's performance on the nonlinguistic flanker task showed a congruency effect (i.e., mean reaction times on the congruent trials were faster than incongruent trials) (see Figure 5(a)). Unlike the usual effects observed on flanker tasks, neutral trials were slower compared to the incongruent trials. Error analysis showed a greater number of errors on the incongruent condition compared to the congruent and neutral conditions as expected in a flanker task (see Figure 5(b)). Cumulative distribution function plots were derived and showed differences across the three conditions only in the range of the 60th to 90th percentile, which was not consistent with the mean RT performance, and showed less congruent RTs compared to neutral RTs and less neutral RTs compared to incongruent RT conditions (see Figure 5(c)). The trend of slower incongruent trials compared to congruent trials was also observed at the 5th percentile (fast trials) level.

**Figure 6:**
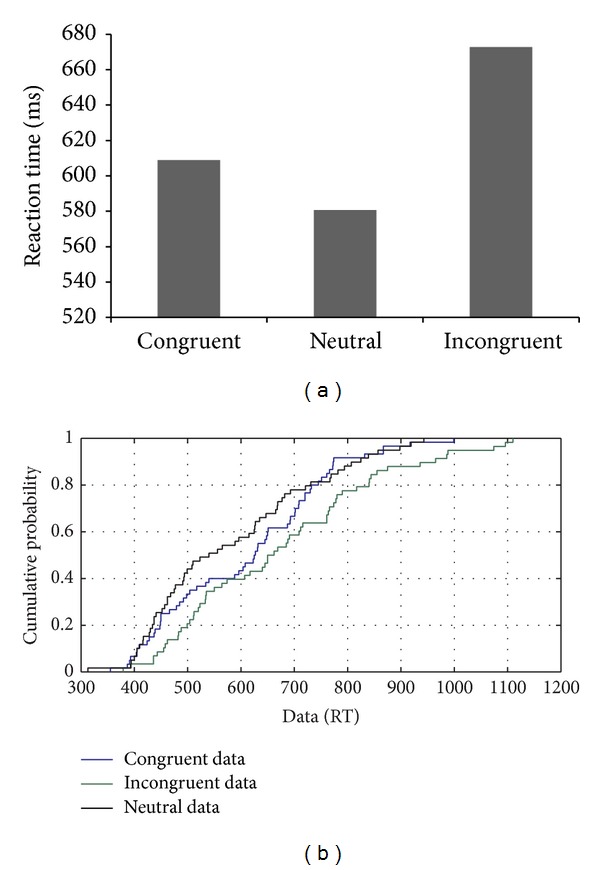
(a-b): Reaction time data and CDF plot based on the performance of MMH on the flanker task with nonlinguistic stimuli.

MMH showed a 98.8% accuracy on the standard flanker task, demonstrating the expected congruency effect with faster RTs on congruent trials compared to the incongruent trials. According to the CDF analysis, a congruency effect was observed with respect to the neutral condition only on slow trials, indicating the involvement of reactive control (see Figures 6(a) and 6(b)). The CDF plots indicated that RTs for the neutral condition were faster than the incongruent condition throughout the distribution, which is suggestive of the presence of an interactive and efficient inhibitory control mechanism.

**Figure 7:**
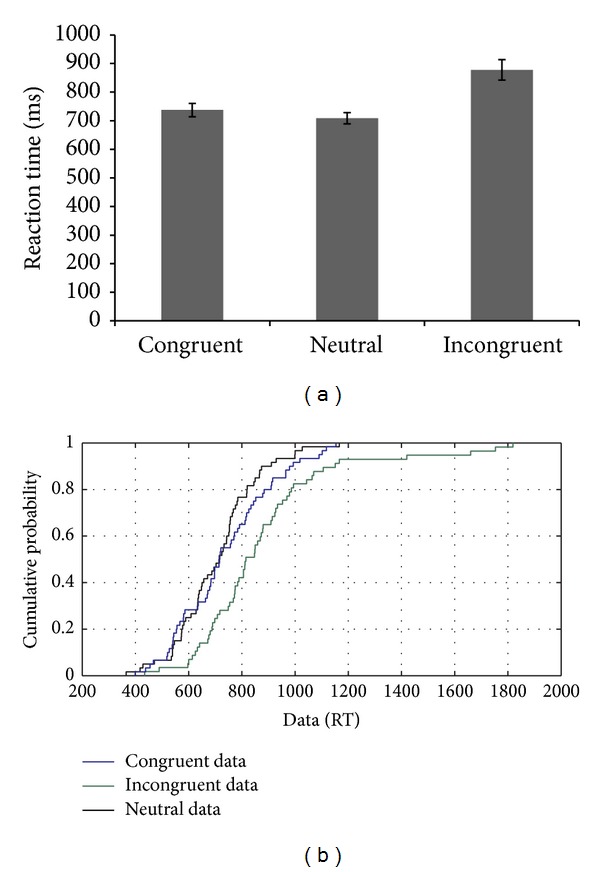
(a-b): Reaction time data and CDF plot based on the performance of SC on the flanker task with nonlinguistic stimuli.

SC demonstrated a 67.2% accuracy on the nonlinguistic flanker task. However, all the errors were made on the incongruent trials. Thus, the flankers' identity was influencing judgment more than the target's identity on the incongruent trials (see Figure 7(a)). The flanker effect was observed with slower RTs on incongruent trials compared to the congruent trials. RTs on incongruent trials were also compared with congruent and neutral conditions. And the RT distributions showed a uniform difference across conditions throughout the distribution (see Figure 7(b)). These results indicated that SC showed no difference between the slow versus fast trials on any of the conditions, demonstrating that both the proactive and reactive control mechanisms contributed to the flanker effects.

**Figure 8:**
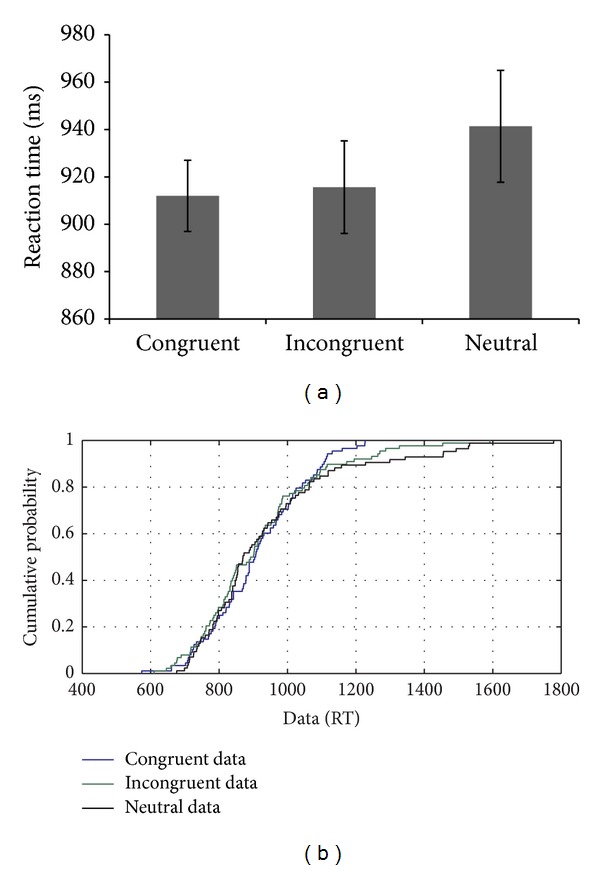
(a-b): Reaction time data and CDF plot based on the performance of MU on the flanker task with nonlinguistic stimuli.

MU's performance on the nonlinguistic flanker task showed a congruency effect with respect to the mean RTs, although his performance on the neutral condition was exceptionally slow compared to the incongruent trials (see Figure 8(a)). CDF analysis showed that the congruency effect was absent (showing no difference between congruent and incongruent trials) on slow trials (i.e., 95th percentile and above), suggesting the involvement of proactive control mechanisms in the efficient performance, which was also highlighted by a high accuracy throughout the distribution (see Figure 8(b)). Uniformity was also observed in the distribution, which changed only in the slow trials, where the distribution shifted to its usual trend of differences across conditions.

Thus, performance on the flanker task with nonlinguistic stimuli showed a similar involvement of the reactive and proactive control mechanisms, contributing to the flanker effects for all four participants. All of the participants similarly showed conflict resolution and executive control effects on slow and fast trials, indicating the efficiency of the control processes in current trial inhibitory effects with nonlinguistic stimuli.

### 3.3. Flanker Task with Linguistic Stimuli

All of the participants performed the flanker task with linguistic stimuli with a fair amount of accuracy. Flanker effects with respect to the reaction times and accuracy on congruent and incongruent trials for L1 and L2 were observed, and the patterns of these effects on slow and fast trials were examined based on the CDF plots.

**Figure 9:**
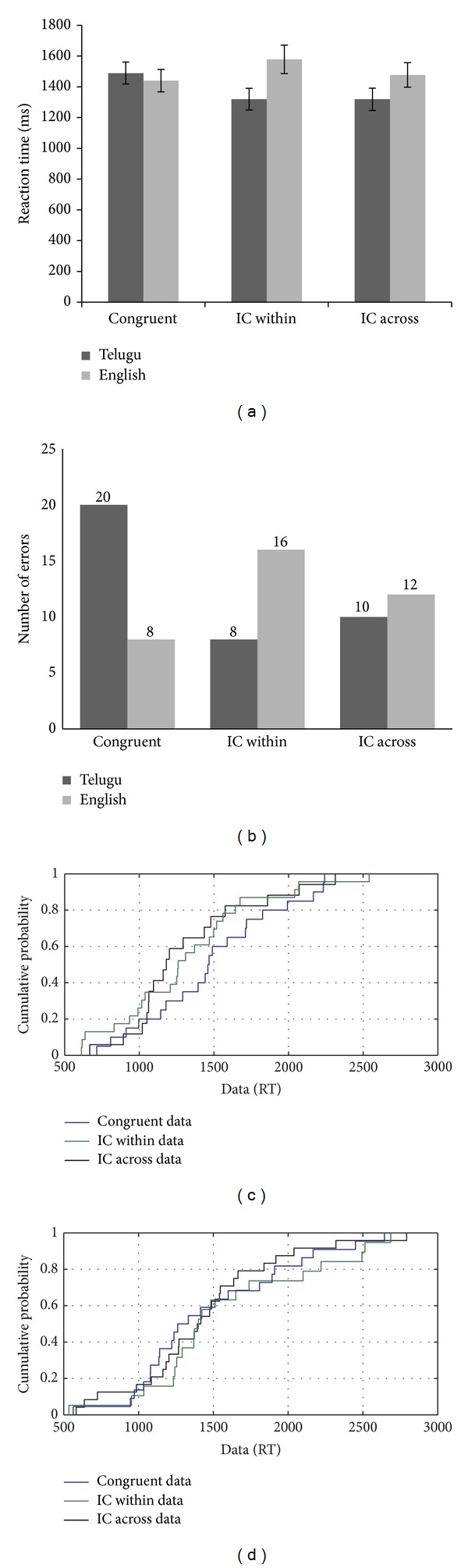
(a–d): Reaction time data, error analysis, and CDF plot based on the performance of CR on the flanker task with linguistic stimuli.

CR's performance on the linguistic flanker task showed a congruency effect for L2, whereas there was an absence of the congruency effect for L1 (see Figure 9(a)). The overall errors across all the conditions were greater for L1 compared to L2. L1 showed errors mostly on the congruent trials, whereas L2 showed a greater number of errors on the incongruent trials (see Figure 9(b)). For the language incongruent condition, the congruency effect was observed only for L2 throughout the distribution (see Figure 9(c)). Both L1 and L2 showed a flanker effect on the language incongruent condition on the fast (5th–20th percentile) and slow (70th–95th percentile) trials. Interestingly, similar patterns for the congruency effect for both L1 and L2 on the cross language incongruent condition were observed. The discrepancy in the mean scores for L1 versus L2 with respect to the congruency effect is suggestive of different underlying processes operating for L1 compared to L2. However, this difference was not explained by the CDF plots, which showed similar patterns of performance on the slow and fast trials for both languages (see Figures 9(c) and 9(d)).

**Figure 10:**
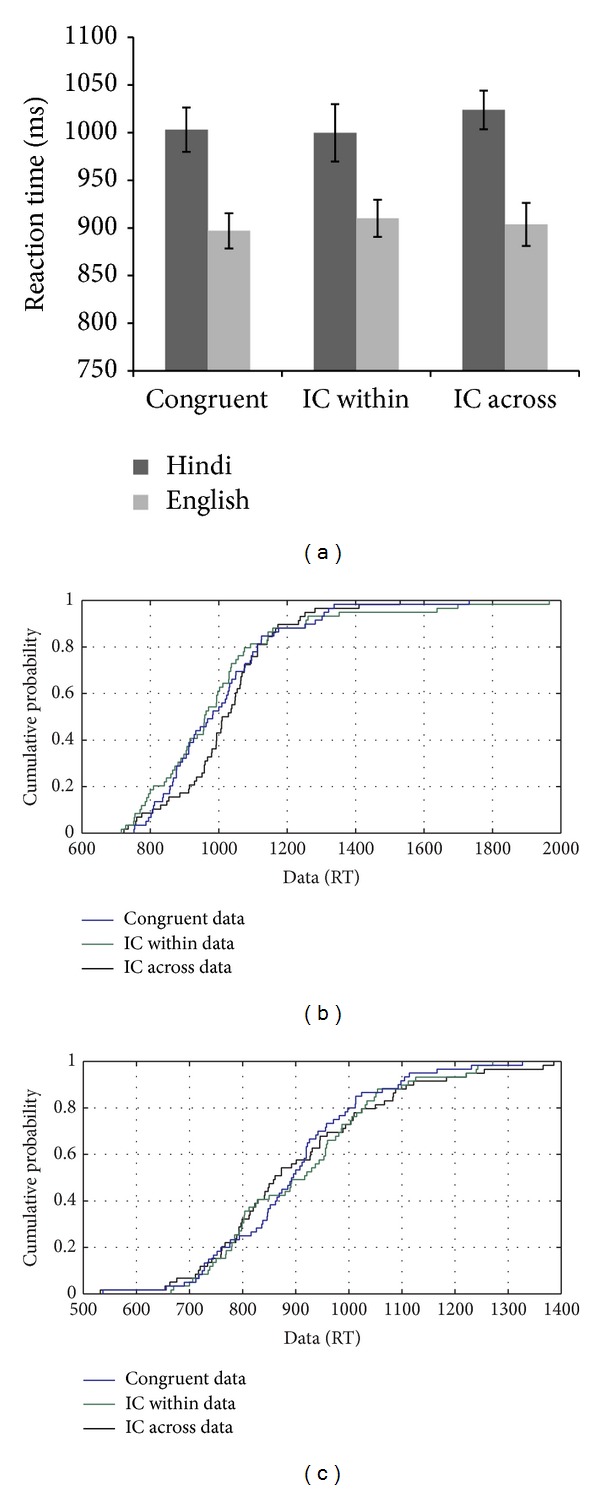
(a–c): Reaction time data and CDF plot based on the performance of MMH on the flanker task with linguistic stimuli.

MMH's performance on the linguistic flanker task showed a congruency effect for both types of incongruent conditions (i.e., IC within and IC across) for L2, whereas for L1, the flanker effect was absent in the within language condition (see Figure 10(a)). The CDF plots showed no difference in the pattern of RT distributions across the three conditions for L2, whereas for L1, the across language incongruent trials showed a congruency effect between the 20th and 70th percentile, which was not observed for the within language incongruent condition (see Figure 10(b)). The congruency effect for the within language incongruent trials was observed only on the slow trials (see Figure 10(c)).

**Figure 11:**
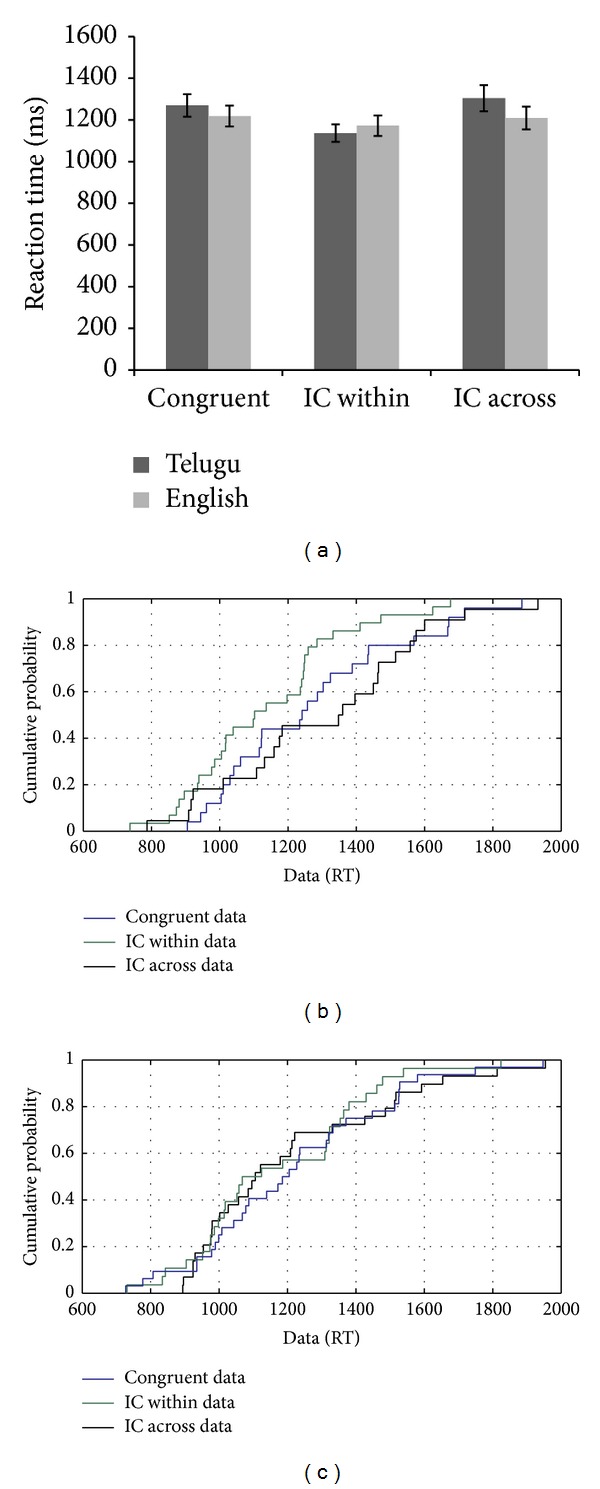
(a–c): Reaction time data and CDF plot based on the performance of SC on the flanker task with linguistic stimuli.

Unlike his performance on the flanker task with nonlinguistic stimuli, SC demonstrated a higher overall accuracy on the linguistic flanker task (92.69%), which supports our assumption with respect to his performance on the previous task; that is, the errors were not due to difficulties in the response selection. Although the differences in the mean reaction times were very small (see Table 4), there was a congruency effect for L1 only on the across language incongruent trials. However, the RT distributions of L1 showed an absence of a congruency effect on the fast trials for the across language incongruent condition (see Figure 11(b)). These results indicated that the interference caused by the flankers in L2 while attending to the target in L1 was resolved using proactive control mechanisms because the effect was not sustained throughout the distribution and was only present for the fast trials. For L2, CDF analysis showed a congruency effect on the within language incongruent condition, but only at the 5th percentile level (fast trials) and was not observed throughout the distribution (see Figure 11(c)). These results indicated a greater dependence on proactive control mechanisms because the difference between the congruent and incongruent trials within a particular language (L2) surfaced only on the fast trials.

**Figure 12:**
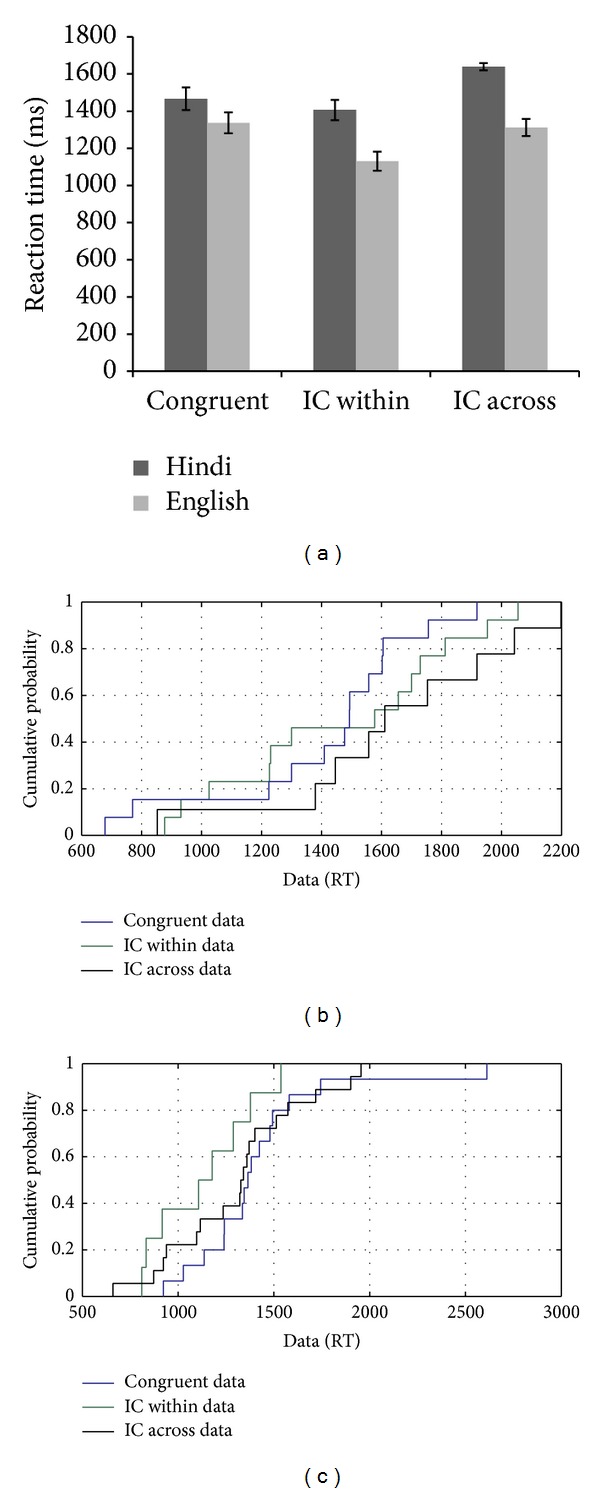
(a–c): Reaction time data and CDF plot based on the performance of MU on the flanker task with linguistic stimuli.

MU's performance on this task showed a congruency effect for L1 and not for L2 with respect to the mean reaction time data (see Figure 12(a)). The flanker effect was present for L1 for the across language incongruent condition and not for the within language incongruent condition. These results indicated that the interference experienced was less when the flankers and the target were from the same language compared to the bilingual trials. Visual inspection of the CDF plots showed the presence of a congruency effect on the slow and fast trials, and these effects were absent only in the range of the 15th–50th percentile. CDF analysis of the across language congruency effect in L1 showed the presence of a flanker effect throughout the distribution. CDF plots showed slowing on the across language incongruent condition compared to the congruent condition for L2 with RTs ranging from 75th to 95th percentiles (see Figure 12(b)). These results indicated an involvement of reactive control mechanisms in a more demanding situation where one needs to inhibit the flankers in L1 to attend to the targets in L2.

Thus, results based on the linguistic flanker task with respect to the within language and across language flanker effects for L1 and L2 indicated a greater variability in performance across the four participants as well as for each participant for L1 versus L2. Our results clearly show that in the case of bilingual language control, bilingual individuals with aphasia appear to show differences in the patterns of performance for L1 versus L2 as well as the recruitment of control mechanisms in resolving conflicts with linguistic stimuli. In addition, the flankers also greatly influenced inhibitory control processes compared to target processing of linguistic stimuli. Thus, it would be equally important to examine suppression-related mechanisms among individuals with bilingual aphasia to investigate the activation-related mechanisms for languages affected in an individual with bilingual aphasia.

### 3.4. Correlation Analysis (Language History Variables and Performance on Control Tasks)

Correlations were determined to examine the relationship between bilingualism-related factors, such as language use and self-rated language proficiency with the experimental task performance across the four participants.A bivariate correlation analysis was performed using two sets of variables: those related to the language background information (language use in L1 and L2, overall language proficiency in L1 and L2, proficiency in speaking, and understanding domain) and those pertaining to the control tasks (flanker effect for L1/L2 in the within language incongruent condition, flanker effect for L1/L2 in the across language incongruent condition, flanker effect for nonlinguistic stimuli, and a positive priming effect and negative priming effect on the negative priming task).

Language use did not show a significant correlation with performance on any of the control tasks. However, interesting trends were observed with respect to the relationship between L1 and L2 proficiency and control tasks, and specifically with linguistic stimuli. Language proficiency in L1 was negatively correlated with the flanker effect of L2 in the within language incongruent condition (*r* = −.970, *p* = .03), whereas it was positively correlated with the flanker effect of L2 in the across language incongruent condition (*r* = .979, *p* = .02). L2 proficiency showed a negative correlation with the flanker effect of L1 and L2 across language incongruent condition (*r* = −.986, *p* = .01 and *r* = .964, *p* = .03, resp.). However, L2 proficiency was positively correlated with L2 within language incongruent condition (*r* = .977, *p* = .02). The observed correlations indicated that the relationship between proficiency and the control task performance among aphasic individuals emerged mostly in bilingual competition on the across language incongruent condition on the linguistic flanker task. When L1 proficiency is low or when L1 is the affected language in aphasia, the flanker effect would also be less on the across language incongruent condition in L2 because the competition/conflict from the weaker L1 flankers would be less. Second language proficiency has been reported to be enhanced compared to L1 by all participants. The negative correlation between L2 proficiency and the flanker effect for L1 and L2 on bilingual trials manifested differently across participants based on individual data. For instance, CR showed a negative correlation in terms of a better L2 proficiency and reduced flanker effect for L1 on the L1 across language incongruent condition. However, for SC and MU, a lower L2 proficiency was correlated with greater flanker effects for L1 in the L1 across language incongruent condition. There was a near significant negative correlation between proficiency in the speaking/understanding domain in L1 and an inhibitory effect (*r* = −.919, *p* = .08) on the nonlinguistic negative priming task. These results suggested that the inhibitory effects on a nonlinguistic negative priming task might increase in lower L1 proficiency. This suggested a potential relationship between L1 proficiency and domain general inhibitory control.

Thus, results based on the correlation analysis suggested that a weaker or affected language in bilingual individuals with aphasia was not correlated with flanker effects in the weaker language compared to the L2 proficiency, which showed a significant relationship with flanker effects in L1 and L2. Inhibitory effects in L1 and L2 surfaced in bilingual competition and are more closely related to proficiency, particularly in the less affected language, which is L2 in most of the participants in the current study. These interesting trends in the current data should be further tested using a larger number of bilingual individuals with aphasia.

To summarise our results, all participants showed the presence of a facilitation effect, in the absence of an inhibitory effect (except for SC) on the negative priming task. CDF analysis showed the presence of an inhibitory effect only on the slow trials for CR, SC, and MU. SC also demonstrated inhibitory effects on fast trials. The flanker task with linguistic and nonlinguistic stimuli showed varying effects across the four participants. A congruency effect was evident on the nonlinguistic flanker task for all the participants with respect to the mean reaction times. CDF analysis revealed interesting patterns of performance. CR and SC showed a congruency effect throughout the distribution, whereas MMH showed a congruency effect only on the slow trials. Conversely, MU showed a congruency effect only on the fast trials. Thus, a rather complex picture emerged from the linguistic version of the flanker task, based on the mean reaction time data. A congruency effect was observed for L2 (i.e., while comparing the congruent condition with the incongruent within language and incongruent across language conditions) only for CR and MMH. However, SC and MU showed a congruency effect only for L1 compared to the congruent condition with the incongruent across language condition. CDF plots also showed varying patterns of performance across participants on the cross linguistic flanker task. CDF plots for L1 (compared to the congruent condition with the within language incongruent condition) showed an absence of a congruency effect except for MMH who showed a congruency effect on slow trials. Interestingly, the congruency effects for L1 (i.e., congruent condition versus incongruent across language condition) throughout the RT distribution of CR and MU on slow trials were observed. All participants showed different patterns of performance in their L2. Error analysis helped to understand the within subject variability in reaction times. However, the highly accurate performance of all the participants in different tasks limited our ability to draw any commonality among them.

## 4. Discussion

The findings of the current study are consistent with the view that the acquisition of another language involves an adaptation to an existing network. Different languages are represented in shared brain regions with common organising principles [36]. Specific patterns of deficits reflect problems of control rather than deficits of pure linguistic origin. Inferences drawn from deficits involve reverse extrapolation to a premorbid state of functioning. An influential aspect of this approach is that patterns of performance (both intact and impaired) suggest selective damage to one or more components or processing pathways. The results of the current study suggest that although inhibitory control underlying selective attention may be impaired in participants with anterior aphasia. The ability to differentiate the target from the distracter may be preserved; thus, the presence of flanker effects in the flanker task. The flanker task and negative priming task are dependent on different processing mechanisms. The presence of positive priming in the absence of negative priming with respect to the RT data observed in our participants is suggestive of the dual route involved in the negative priming task. It has been postulated that positive priming is strongly affected by perception in contrast to negative priming, which emerges during selection [37]. We have found that such dissociations between positive and negative priming effects in the current study suggest difficulties with respect to selection as a component of control processes among bilingual individuals with aphasia.

Reactive and proactive control mechanisms underlying the performance on each task for each participant were explored using CDF analysis. All four participants showed a dependence on the reactive control mechanisms with specific variations observed between the two languages that were known by each of the four participants. For example, CR's congruency effect on the linguistic flanker task showed an interesting language specific variation. L2 (English) showed the involvement of reactive control, whereas L1 showed a reliance on proactive control mechanisms. MMH showed an L1 congruency effect only when L1 was flanked by L2 on slow trials, suggesting the involvement of reactive control. These effects were similar to those observed in CR's performance. An interesting observation was that MU showed greater interference from the same language (when flankers were in the same language as the target) compared to the condition that involved across language competition. However, this effect was only observed for L2, whereas L1 showed the congruency effect in across language conflict. The nonlinguistic flanker task showed the involvement of both proactive and reactive control mechanisms, except for MMH and MU. MMH showed an involvement of the reactive control mechanism and MU showed a reliance on proactive control. These two mechanisms are not mutually exclusive. Thus, it is possible that CR and SC showed the involvement of both mechanisms to resolve the conflict for efficient performance. CDF plots suggested that the magnitude of the effects was larger for facilitation or the positive priming effect on slow trials, and differences between the control condition and ignored repetition condition were more prominent on fast trials for MMH and MU on the negative priming task. In both cases, it is probable that the sustained activation of all four items (2 pictures from the prime trial and 2 from the probe trial) resulted in the slowing of the response in the control condition, due to a greater interference from unattended stimuli. There was an interesting dissociation observed in CR's performance, demonstrating an involvement of proactive control during facilitation and reactive control for inhibition. Such a tradeoff may be due to dual mechanisms involved in facilitation versus inhibition. The distinction between proactive and reactive control is useful in elucidating the variations in cognitive control mechanisms due to the influences from bilingualism-related factors, which need to be explicitly manipulated and examined in future research. As a result of their limited processing resources, the effective engagement of proactive control may be problematic for individuals with aphasia and may thus engage the reactive control mechanisms, which do not require the individual to sustain control over an extensive period of time [27].

Another interesting area to explore is the interaction of bilingual language control and general purpose cognitive control and thus, we compared the performance on linguistic and nonlinguistic flanker tasks. Performance-based differences were evident on flanker tasks with nonlinguistic versus linguistic stimuli. Interestingly, the variations in the performance of each participant surfaced to a greater degree in the linguistic stimuli for both L1 and L2. Except for CR, all of the other participants showed differences in their performance between the two tasks. For example, more reliance on reactive control mechanism in the performance of MMH on the nonlinguistic flanker task was observed, whereas the proactive control was predominant in the across language incongruent condition on the linguistic flanker task. This trend was reversed for MU.

Results obtained from the current study helped to form the stage for further studies to enhance our understanding of language control and cognitive control in bilingual aphasia as well as to improvise the rehabilitation process. This is supported by the fact that therapy in L2 was related to a better performance in L2 on the linguistic flanker task (in the case of CR and MMH), while therapy focusing predominantly on L1 resulted in a better performance in L1. Such a domain specific effect of therapy was also reported in a study by Abutalebi et al. [2], where improvement in the naming performance resulted in an improvement in the naming network only. Abutalebi and colleagues [2] also discussed the dissociation between the naming and control pathways, which was consistent with the observations of the present study with respect to the variations in performance between linguistic and nonlinguistic control tasks.

We also observed that individuals with better scores on the WAB did not show an involvement of the proactive control mechanism with the data based on the negative priming task. Thus, there is a need to perform both linguistic and nonlinguistic control tasks while profiling individuals with bilingual aphasia. It is possible that individuals with bilingual aphasia may respond to speech and language therapy and show an improvement in language skills in the affected language, but may still demonstrate problems with executive control. Another interesting relationship between subjective information (see Tables 1, 2, and 3) and task performance was via premorbid language use (in percentage) and the linguistic flanker task. Premorbid language use was the same for SC and MU, whereas for CR and MMH, L1 was the dominant language. This was translated to the performance on linguistic flanker task, where language was dominant premorbidly and was affected compared to the other language (in these cases L2). SC and MU with a similar dominance of language use premorbidly, showed an absence of the flanker effect in both languages. In contrast to CR and MMH, their L1 performance was better than L2. Although such links between language use and task performance are interesting, the extrapolation of such findings via only single case studies should be carefully performed. However, the descriptive account of language use and task performance shows a relationship between the two variables, but the correlation analysis did not show a statistically significant correlation with the performance on any of the control tasks, which could be due to the variance across participants with respect to language use.

Studies investigating the interaction between bilingualism and control processes have theoretical and clinical implications. The case study approach employed in the current study provided an individually specific profile of bilingual individuals with aphasia with respect to cognitive control processes and the nature of control mechanisms, which may influence the recovery patterns and could thus be considered during the rehabilitation process. This study also highlighted that the performance of no two bilingual aphasics was the same and thus required a detailed assessment of both language control and cognitive control processes particularly relevant for individuals with bilingual aphasia. It has been reported by Abutalebi and Green [2] that the effect of treatment in bilingual aphasia was dependent on the integrity of naming and control pathways, indicating the need to address both linguistic and control systems. Apart from providing insight into language control and cognitive control mechanisms, such a profile of individuals with aphasia may help to decide the language for therapy in bilingual aphasia. Although, the current data are limited in establishing such a claim, they open new avenues of research. Performance on the flanker task and negative priming task may indicate the use of selective language therapy or bilingual language therapy based on the level of interference. Apart from the treatment decisions, clinical implications of language control and cognitive control mechanisms may act as a main determinant of cognitive-linguistic recovery in aphasia [2].

Taken together, the variations observed in the performance of each participant across tasks and stimuli strongly suggested that there is dissociation between bilingual language control and general purpose cognitive control mechanisms. These observations were further strengthened by the findings based on the correlation analysis between bilingualism-related factors (language use and proficiency) and performance on control tasks, which showed that the relationship between proficiency and inhibitory effects in L1 and L2 surfaced primarily in case of bilingual competition. L1 proficiency with respect to the speaking/understanding domain was correlated with a sustained inhibitory control (negative priming effect with nonlinguistic stimuli) and L2 proficiency was correlated with cross-linguistic flanker effects for both L1 and L2, indicating a dissociation between the role of L1 versus L2 proficiency in domain general cognitive control and bilingual language control, respectively. These interesting trends in the current data need to be empirically tested further with explicit manipulations related to L1 and L2 proficiency using a larger group of individuals with bilingual aphasia and their performance on a range of control tasks.

## 5. Conclusion

The present study was designed to examine the performance of bilingual aphasics on executive control tasks that test the circuits implicated in language control and cognitive control. CDF analysis was a promising tool used to examine the variations in performance within and across individuals, tasks and stimuli. Current trial inhibitory effects were observed among individuals with bilingual aphasia, whereas a sustained inhibitory control (as assessed on the negative priming task with nonlinguistic stimuli) was found to be compromised. Interestingly, sustained inhibitory control was correlated with L1 proficiency. All the participants demonstrated the use of reactive control mechanisms to compensate for the limited resource system. We also found differences in the involvement of control mechanisms for linguistic stimuli between L1 and L2 with L1 depending more on proactive control and L2 depending more on the reactive control mechanisms. Importantly, these mechanisms were not mutually exclusive but interacted for efficient inhibitory control. The observations of the current investigation involved a series of four case studies, which provided valuable insight into the nature of the control mechanisms and were not limited to the task performance and deficits in cognitive abilities. A longitudinal study on individuals with bilingual aphasia helped to monitor the changes in cognitive control (which also appeared to be affected among bilingual aphasics). Control processes, such as selection, inhibition, and monitoring particularly sustained inhibitory control, appear to serve as the underlying resource systems for bilingual language control.

## Figures and Tables

**Table 1:** Language background information based on current state (poststroke aphasia data).

Participants	CR	MMH	SC	MU
Languages exposed at home	Telugu (sometimes Kannada)	Urdu/Hindi	Telugu (sometimes Tamil with extended family)	Hindi/Urdu

Languages exposed at office/workplace/college	English, Kannada, Hindi	English, Hindi, Kannada	English, Telugu, Hindi	English, Hindi

Age of acquisition:	L1 (Telugu): since birth	L1 (Hindi/Urdu): since birth	L1 (Telugu): since birth	L1 (Hindi/Urdu): since birth
	L2 (English): 10th standard	L2 (English): 3.5 years	L2 (Tamil): exposed since birth	L2 (English): since school that is 1st standard
	L3 (Kannada): after arriving at Kannada speaking state due to occupational needs in 2008	L3 (Kannada): after arriving at Kannada speaking state (10 years)	L3 (English): since school that is 1st standard	

Order of dominance (premorbid):				
L1	Telugu (60%)	Hindi/Urdu (70%)	Telugu (50%)	Hindi (50%)
L2	English (40%)	English (30%)	English (50%)	English (50%)

Order of dominance (postmorbid):				
L1	Telugu (30%)	Hindi/Urdu (85%)	Telugu (60%)	Hindi (90%)
L2	English (70%)	English (15%)	English (40%)	English (10%)
	Sporadic usage of Kannada and Hindi	Sporadic usage of Kannada	Sporadic usage of Tamil and Hindi	

Modality of language acquisition:				
L1	both (oral/written and formal/informal)	both (oral/written and formal/informal)	both (oral/written and formal/informal)	both (oral/written and formal/informal)
L2	both (oral/written and formal/informal)	both (oral/written and formal/informal)	both (oral/written and formal/informal)	both (oral/written and formal/informal)

Family members uses following languages:				
Grandparents, parents, siblings-	Telugu	Hindi/Urdu	Telugu/Tamil	Hindi
Neighbours/children-	Kannada	Kannada	Hindi/Telugu	Hindi

Language use choice: 3 point rating (composite scores)	(can perform 3/10 tasks)	(can perform 6/10 tasks)	(can perform 6/10 tasks)	(can perform 5/10 tasks)
L1	3	1	1	2.7
L2	2	2.7	3	2

Language proficiency 5 point rating (composite scores)	(can perform 5/15 tasks)	(can perform 7/15 tasks)	(can perform 6/15 tasks)	(can perform 10/15 tasks)
L1	2.25	2.53	3.1	4.25
L2	4.25	3.25	2.83	2.25

Self-reported proficiency (5-point rating)				
Reading (L1 L2)	4 4	4 4	2 2	2 3
Writing (L1 L2)	3 4	3 4	1 2	2 2
Speaking (L1 L2)	3 4	3 4	2 2	3 2
Understanding (L1 L2)	4 4	4 4	4 3	4 4

**Table 2:** Demographic information.

Participants	CR	MMH	SC	MU
Age	33 years	34 years	35 years	59 years
Etiology	Bacterial meningitis	CVA	Trauma	CVA
Time post stroke	17 months	26 months	15 months	20 months
Native language	Telugu	Hindi/Urdu	Telugu	Hindi
Educational and work background	MBA and currently employed as a banker	Postgraduate and currently unemployed	B. Tech and own a construction business	Retired as assistant controller of examination for an university
Languages known (in order of dominance)	Telugu, English, Hindi	Hindi, English, Urdu	Telugu, English, Tamil, Hindi	Hindi, English, Urdu
Aphasia type	Anomic aphasia	Anomic aphasia	Broca's aphasia	Anomic aphasia
Rehabilitation period	15 months	20 months	3 months	17 months
Aphasia severity	2	3	1	3
Language for therapy	L2	Both L1 and L2	Both L1 and L2, more emphasis L1.	L1

**Table 3:** Scores on the Western Aphasia battery.

Participants WAB task (maximum scores)	CR	MMH	SC	MU
Spontaneous speech				
Information content (10)	7	9	4	8
Fluency (10)	4	9	5	9
Auditory verbal comprehension				
Yes/no question (60)	48	60	20	58
Auditory word recognition (60)	60	58	53	48
Sequential commands (80)	40	72	21	74
Repetition (100)	45	81	26	79
Naming				
Object naming (60)	40	59	4	45
Word fluency (20)	3	12	2	15
Sentence completion (10)	4	7	2	8
Responsive speech (10)	3	10	5	9

**Table 4:** Mean reaction time and standard deviations on control tasks.

Participants	CR	MMH	SC	MU
Flanker task (nonlinguistic)				
Congruent	964.42	608.85	737.2	911.98
	(556.89)	(153.17)	(181.92)	(140.86)
Incongruent	1183	580.62	877.63	915.65
	(767.47)	(158.50)	(269.85)	(183.13)
Neutral	1221.61	672.70	708.56	941.3
	(597.01)	(186.53)	(153.81)	(217.62)
Flanker task (linguistic)				
L1 congruent	1488.6	1003.08	1269.2	1467
	(450.91)	(178.79)	(270.48)	(381.61)
L1 incongruent within	1319.52	999.71	1136.62	1406.38
	(476.38)	(231.41)	(229.28)	(342.35)
L1 incongruent across	1318.94	1023.84	1303.63	1639.66
	(423.27)	(154.55)	(296.71)	(387.03)
L2 congruent	1439.38	896.9	1218.21	1336.71
	(503.12)	(143.40)	(283.56)	(380.55)
L2 incongruent within	1578.68	910.13	1171.92	1130.87
	(569.45)	(149.55)	(256.05)	(252.92)
L2 incongruent across	1476.91	903.64	1208.93	1312.33
	(526.39)	(173.09)	(292.72)	(340.48)
Negative priming task				
Attended repetition	1651.69	607.45	648.08	832.27
	(769.31)	(117.23)	(301.44)	(284.72)
Control	1939.81	933.05	791.27	1626.32
	(681.08)	(131.70)	(334.3)	(269.32)
Ignored repetition	1751.57	807.33	1091.83	1232.01
	(732.14)	(201.76)	(554.43)	(434.35)

**Table 5:** Rubric for picture description: for spoken discourse analysis.

Strong: 3 points	Average: 2 points	Weak: 1 point
Overall impact and achievement of purpose		
**3** Presents a vivid, memorable picture of a person, place or things	**2** Presents a clear picture of a person, place, or thing	**1** Presents an unclear or confusing picture of a person, place and thing
**3** Establishes a dominant, or main, impression of the picture	**2** Focuses on important characteristic(s) of the picture	**1** Presents an unfocused array of characteristics of the picture
**3** Conveys a clear sense of purpose	**2** Suggests the speakers purpose	**1** Unclear or inadequate indication of speakers' purpose

Organization and techniques		
**3** Uses a clear, consistent method of organization of event	**2** Method of organization is usually clear and consistent	**1 **Method of organization is difficult to identify or follow
**3** Coherence and cohesion demonstrated through some appropriate use of devices (transitions, pronouns, causal linkage, etc.)	**2** Coherence and cohesion (sentence to sentence) evident; may depend on holistic structure, most transitions are appropriate	**1** Evidence of coherence may depend on sequence. If present, transitions may be simplistic or even redundant

Mechanics		
**3** Very few, if any errors in grammar and pronunciation and presence of few pauses (filled and unfilled)	**2** Small number of errors in grammar and pronunciation and presence of indefinable pauses (filled and unfilled)	**1 **Numerous errors in grammar and pronunciation and presence of pauses (filled and unfilled)

Note: a composite score on the picture description task is the sum of ratings across the three aspects of discourse analysis.
